# The Long Noncoding RNA *LINC00963* Inhibits Corneal Fibrosis Scar Formation by Targeting *miR-143-3p*

**DOI:** 10.1089/dna.2021.1034

**Published:** 2022-04-19

**Authors:** Lixia Zhang, Jinning Gao, Anjing Gong, Yanhan Dong, Xiaodan Hao, Xuekang Wang, Jian Zheng, Wenmeng Ma, Yiying Song, Jie Zhang, Wenhua Xu

**Affiliations:** ^1^ Department of Inspection, The Medical Faculty of Qingdao University, Qingdao, China.; ^2^ Department of Clinical Laboratory, The Affiliated Hospital of Qingdao University, Qingdao, China.; ^3^ Center for Molecular Genetics, Institute for Translational Medicine, Qingdao University, Qingdao, China.; ^4^ Department of Neurosurgery, The Affiliated Hospital of Qingdao University, Qingdao, China.

**Keywords:** long noncoding RNAs, cornea, fibrosis, microRNA, transforming growth factor

## Abstract

Corneal fibrosis is a complication of severe corneal injury, one of the major causes of vision loss. The formation of myofibroblasts has emerged as a key stimulative factor of corneal fibrosis. In the current study, we focused on the role of LINC00963 in regulating corneal fibrosis. Transforming growth factor β1 (TGF-β1) was used to induce human corneal stromal cells differentiating into corneal myofibroblasts, and the significant increase of α-smooth muscle actin (α-SMA) was verified by quantitative real-time PCR (qRT-PCR), western blot, and immunofluorescence, respectively. LINC00963 was identified to be one-half decreased compared with nonstimulated human corneal stromal cells, indicating that it might play a role in corneal fibrosis. Interestingly, overexpression of LINC00963 resulted in decreased formation of myofibroblasts indicating that it might exhibit an inhibiting effect. Moreover, bioinformatics tool was applied to predict the downstream target of LINC00963. We investigated that LINC00963 suppressed α-SMA induced by TGF-β1 in corneal fibroblasts, at least in part, by downregulating the expression of miR-143-3p. In addition, either LINC00963 promotion or miR-143-3p inhibition could significantly decrease myofibroblast contractility and collagen I and III secretion, which are the key to contribute to corneal fibrosis. Taken together, our study identified LINC00963 as a promising therapeutic target.

## Introduction

Excessive healing reaction after corneal injury easily leads to the formation of corneal fibrosis scars, which seriously affects vision (Naito *et al.*, 2014). Transforming growth factor β1 (TGF-β1) stimulating the transdifferentiation of corneal stromal cells into myofibroblasts is considered to be the ultimate reason of corneal scar formation (Wei *et al.*, 2019). At present, there is no effective treatment method for vision loss caused by corneal fibrosis scar formation. Clinically, steroid hormones, antimetabolites, surgical treatment, and other treatment methods are used, all of which have unsatisfactory curative effects and large side effects (Yang *et al.*, 2011; Milani *et al.*, 2013; Dvashi *et al.*, 2014).

Lately, noncoding RNA gene therapies using noncoding RNA have emerged as new therapeutic approach for fibrotic diseases, which are more effective than traditional drugs or antibody therapy (Abdel-Al *et al.*, 2018; Peng *et al.*, 2018; Zhao *et al.*, 2018). Noncoding RNAs are a class of RNAs that rarely encode proteins, which include long noncoding RNAs (lncRNAs), circular RNAs, piwi-interacting RNAs, guide RNAs, microRNAs (miRNAs), etc. lncRNAs are often with more than 200 nucleotides length, which are rarely protein-coding potential and are involved in a variety of pathophysiological processes.

The regulatory mechanisms of lncRNAs are mainly as follows: (a) lncRNAs can bind to DNA methyltransferase (DNMT), thereby inhibiting promoter methylation and promoting gene expression (Qi *et al.*, 2016). (b) lncRNAs can regulate histone modification and chromosome reconstruction. (c) lncRNAs can regulate gene transcription by interacting with transcription factors (Kugel and Goodrich, 2012). (d) lncRNAs act as a precursor of some miRNAs to regulate gene expression (Zou *et al.*, 2016). (e) lncRNAs can act as miRNA sponge and then upregulate the translation of its targeting mRNAs, which is the most common regulatory mechanism (Yan *et al.*, 2015).

MiRNAs are a class of small single-stranded noncoding RNA molecules in length of 18–22 nucleotides. They can regulate gene expression at transcriptional (promoting mRNA degradation by binding to 3′-UTR of the target mRNA) and post-transcriptional (inhibiting protein translation) levels. Studies have reported that dysregulation of noncoding RNA is associated with poor treatment of common ophthalmic diseases (Funari *et al.*, 2013; Huang *et al.*, 2015). So far, lncRNA NEAT1 (Bai *et al.*, 2018), miR-206 (Li *et al.*, 2015), miR-21 (Zhang *et al.*, 2017), miR-145 (Ratuszny *et al.*, 2015), and miR-184 (Park *et al.*, 2017) have been reported to participate in the mechanism of scar formation or neovascularization after corneal fibrosis.

miR-143 showed strong expression in TGF-β1-induced subconjunctival fibrosis, and inhibition of miR-143 expression decreased myofibroblast formation to reverse the fibrosis (Hwang *et al.*, 2019). LINC00963 has been reported to affect renal fibrosis (Chen *et al.*, 2018) and participate in the development of multiple tumors such as osteosarcoma (Zhou *et al.*, 2019), melanoma (Jiao *et al.*, 2018), and hepatocellular carcinoma (Wu *et al.*, 2018). However, no research has shown that it participates in corneal fibrosis. Thus, whether lncRNAs are involved in corneal fibrosis remains to be elucidated.

Recently, lncRNAs have been proposed to be involved in the complex processes, such as fibrosis and scarring. This study focused on LINC00963 to study its role in corneal fibrosis. We also investigated that miR-143-3p, which was associated with cardiac fibrosis (Li *et al.*, 2019), was negatively regulated by LINC00963. Based on our findings, LINC00963 relieved corneal fibrosis by competitively binding to miR-143-3p.

## Materials and Methods

### Cell culture and treatment

Human corneal stromal cell line was gifted from Qingdao Eye Hospital. Cells were maintained in Dulbecco's modified Eagle's media: Nutrient Mixture F-12 (DMEM/F-12) with 10% fetal bovine serum and antibiotics (100 IU/mL penicillin and 100 mg/mL streptomycin) in a humidified atmosphere of 5% CO_2_ at 37°C. This experiment does not involve animal or human experiments and does not require IRB approval.

### Quantitative real-time PCR

Total RNA was extracted from human corneal stromal cells and fibrotic corneas or normal corneas using TRIzol reagent (Invitrogen, Carlsbad, CA) according to the manufacturer's instructions. Reverse transcription (RT) of mRNA and lncRNA to cDNA was synthesized with random primers by PrimeScript RT Reagent Kit (Takara Biomedical Technology, Dalian, China), whereas the reverse primer of miRNA is synthesized by BGI Genomics (Shenzhen, China). The SYBR Premix Ex TaqII Kit (Takara Biomedical Technology) was used for quantitative real-time PCR (qRT-PCR) analysis.

The primers for α-smooth muscle actin (α-SMA), GAPDH, U6, and miR-143-3p are listed in Table 1. All data analyses were operated by qRT-PCR system (Bio-Rad Biosystems). U6 and GAPDH, respectively, served as the internal control for miRNA and mRNA quantification. All qRT-PCR experiments were performed in triplicates.

**Table 1.** Primer Sequences for Quantitative Real-Time PCR

Gene	Primer sequence
α-SMA	Forward: 5′-AGCCAAGCACTGTCAGGAAT-3′
	Reverse: 5′-CACCATCACCCCCTGATGTC-3′
GAPDH	Forward: 5′-ACCACAGTCCATGCCATCAC-3′
	Reverse: 5′-TCCACCACCCTGTTGCTGTA-3′
LINC00963	Forward: 5′-GGTAAATCGAGGCCCAGAGAT-3′
	Reverse: 5′-ACGTGGATGACAGCGTGTGA-3′
GAS5	Forward: 5′-GAAGTCCTAAAGAGCAAGCC-3′
	Reverse: 5′-AGCCGACTCTCCATACCTTT-3′
NORAD	Forward: 5′-AAGCTGCTCTCAACTCCACC-3′
	Reverse: 5′-GGACGTATCGCTTCCAGAGG-3′
U6	Forward: 5′-CTCGCTTCGGCAGCACA-3′
	Reverse: 5′-AACGCTTCACGAATTTGCGT-3′
miR-143-3p	Forward:5′-ACACTCCAGCTGGGTGAGATGAAGCACTG-3′
	Reverse: 5′-TGGTGTCGTGGAGTCG-3′

### miRNA, siRNA reagents, and their application

miRNA agomir or antagomir and siRNA were synthesized by Gene Pharma (Shanghai, China). All the sequences are shown in Table 2. Lipofectamine RNAiMAX (Invitrogen) was used for transfection of miRNAs *in vitro*. Human corneal stromal cells were planted in wells or plates 24 h before use with 40–60% confluence.

**Table 2.** Sequence of Transfection

Gene	Sequence
miR-143-3p-agomir	Sense: 5′-UGAGAUGAAGCACUGUAGCUC-3′
	Antisense: 5′-GCUACAGUGCUUCAUCUCAUU-3′
agomir-NC	Sense: 5′-UUCUCCGAACGUGUCACGUTT-3′
	Antisense: 5′-ACGUGACACGUUCGGAGAATT-3′
miR-143-3p-antagomir	5′-GAGCUACAGUGCUUCAUCUCA-3′
antagomir-NC	5′-UCAUUUUGUGUAGUACAA-3′
si-LINC00963	Sense: 5′-GGUUCCUCAUCUGCCAGUUTT-3′
	Antisense: 5′-AACUGGCAGAUGAGGAACCTT-3′

### Plasmid construction

According to the sequences of LINC00963 in Genebank, plasmid containing full length of LINC00963 and negative control (NC) plasmid were synthesized by BGI (Shenzhen, China).

### Dual-Luciferase Reporter Assay

The wild-type fragment or mutants of the predicted binding site of miR-143-3p on LINC00963 transcript were synthesized and constructed into the pGL3-control reporter vector (Promega, Madison, WI). 293T cells were harvested at 24 h after transfection and assayed for luciferase activity by the Dual Luciferase Reporter Assay Kit (Promega).

### Western blot analysis

Anti-α-SMA antibody and anti-GAPDH antibody were purchased from ABclonal (Wuhan, China), and horseradish peroxidase (HRP)-labeling Goat anti-Rabbit IgG and HRP-labeling Goat anti-Mouse IgG were purchased from EpiZyme (Shanghai, China). RIPA lysis buffer was purchased from Solarbio Life Sciences (Beijing, China). RIPA lysis buffer supplemented with phenylmethylsulfonyl fluoride (Roche) was used to lysis cell or tissue samples. The concentrations of all protein samples were assayed using a BCA Protein Assay Kit (Merck, China). Add equal amounts of protein samples to the gel wells and separate them by 10% SDS-polyacrylamide gel electrophoresis gels.

After electrophoresis and transfer of the protein band to the polyvinylidene fluoride membrane, the membrane was blocked with 5% skim milk dissolved in Tris-buffered saline for 1 h. Then membranes were incubated with the following primary antibodies: α-SMA (1:1000 dilution; ABclonal) and GAPDH (1:50,000 dilution; ABclonal) for 1.5 h at room temperature or shaking at 4°C overnight. Blots were washed thrice for 10 min and incubated with goat-anti-mouse/rabbit secondary antibody (1:3000 dilution; EpiZyme) for 1 h at room temperature. Visualization was performed using Omni-ECL™ Femto Light Chemiluminescence Kit (EpiZyme).

### Immunofluorescence

Immunofluorescence staining was performed on human corneal stromal cells grown in 24-well (ExCell Bio.). Cells were washed using phosphate buffered saline (PBS) thrice and fixed with 4% paraformaldehyde for 10 min, permeabilized with 0.1% Triton X-100 in PBS for 5 min, followed by a blocking step with PBS containing 2% bovine serum albumin for 90 min at room temperature. Subsequently, cells were stained with rabbit anti-human α-SMA antibody for 90 min at room temperature or shaking at 4°C overnight, washed, and incubated with FITC goat anti-rabbit IgG (ABclonal) for 60 min. The slides were mounted in a closure containing DAPI (Solarbio Life Sciences) and placed on a confocal microscope equipped with a FITC or DAPI filter set (using a 63 oil objective).

### Cell contraction assay

Corneal stromal cells were transfected with LINC00963 or miR-143-3p antagomir or NC for 24 h and then stimulated with TGF-β1 for 48 h to differentiate into myofibroblast. Then cells were seeded into collagen gels; after 48 h, gel contraction was assessed according to the protocol of the kitCell (Biolabs, Inc.).

### Collagen secretion assay

Cells were seeded into 48-well plates and further transfected with pcDNA3.1-LINC00963 or miR-143-3p agomir or antagomir for 24 h and then stimulated with TGF-β1 (2 ng/mL) for 48 h. The content of collagen I and collagen III in supernatants was detected by Enzyme-Linked Immunosorbent Assay (ELISA) Kits (Shanghai Jianglai Biotech, Shanghai, China). Optical density was measured at 450 nm.

### Statistical analysis

Data analysis was performed using SPSS 22.0 (SPSS, Chicago) statistical software. The data were represented as $\bar{x}\pm s$, the *t*-test was used to compare two sample variables, and one-way analysis of variance was used to compare the means between groups. *p* < 0.05 was considered to be statistically significant.

## Results

### TGF-β1 promoted human corneal stromal cells to transdifferentiate into myofibroblasts

**FIG. 1.**
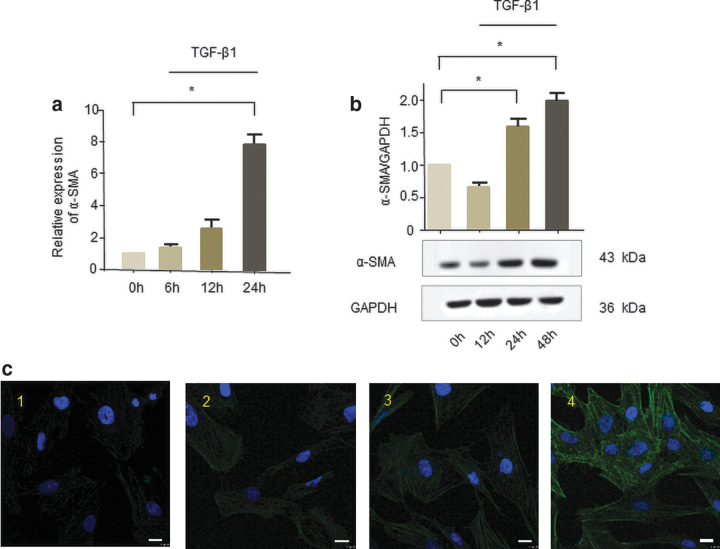
The role of TGF-β1 in promoting the transdifferentiation of human corneal stromal cells into myofibroblasts *in vitro*. Human corneal stromal cells were treated with TGF-β1 (2 ng/mL) for 6, 12, and 24 h. **(a)** qRT-PCR was used to assess α-SMA mRNA levels, and GAPDH worked as internal control. (*n* = 4, *p < 0.05) Human corneal stromal cells were treated with TGF-β1 (2 ng/mL) for 12, 24, and 48 h. **(b)** Western blot was used to assess α-SMA protein levels, and GAPDH was used as internal control. **(c)** Representative immunofluorescence pictures. Transdifferentiated myofibroblast cells have relatively large volume and high fluorescence intensity than human corneal stromal cells. Cells were stained with DAPI to visualize the nucleus (*blue*), and anti-α-SMA-antibody conjugated to FITC to visualize α-SMA (*green*) as marker for myofibroblast differentiation (1:0 h; 2:12 h; 3:24 h; 4:48 h; scale bars = 10 μm). *p < 0.05. α-SMA, α-smooth muscle actin; qRT-PCR, quantitative real-time PCR; TGF-β1, transforming growth factor β1. Color images are available online.

Human corneal stromal cells were treated with 2 ng/mL TGF-β1 for 6, 12, and 24 h, respectively. *De novo* expression of α-SMA, a widely accepted myofibroblast marker (Tomasek *et al.*, 2002), showed that human corneal stromal cells successfully transdifferentiated into myofibroblasts (Fig. 1a–c).

### Candidate lncRNAs involved in scarring of the cornea

**FIG. 2.**
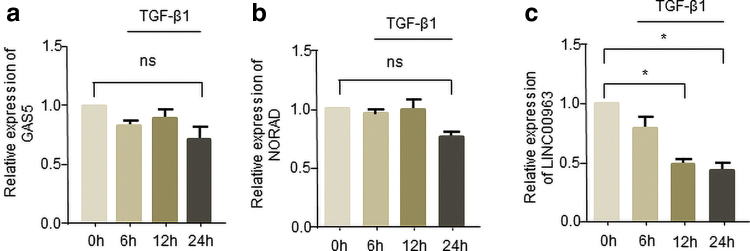
Candidate lncRNAs potentially involved in corneal fibrosis. **(a–c)** Measurement of GAS5, NORAD, and LINC00963 levels in human corneal stromal cells treated with TGF-β1 (2 ng/mL) at the indicated times. *p < 0.05. lncRNA, long noncoding RNA. Color images are available online.

Through extensive literature searches, we have discovered some lncRNAs that may play a role in fibrosis, such as GAS5 (Tao *et al.*, 2017), NORAD (Kawasaki *et al.*, 2018), and LINC00963 (Chen *et al.*, 2018). We detected the expression of candidate lncRNAs in human corneal stromal cells treated with TGF-β1 in a time-dependent manner. The results showed that GAS5 (Fig. 2a) and NORAD (Fig. 2b) had less change under the stimulation of TGF-β1. However, LINC00963 expression was gradually decreased after TGF-β1 treatment (Fig. 2c), indicating that LINC00963 may be involved in the occurrence of corneal fibrosis.

### Overexpression of LINC00963 prevented human corneal stromal cells from transdifferentiating into myofibroblasts

**FIG. 3.**
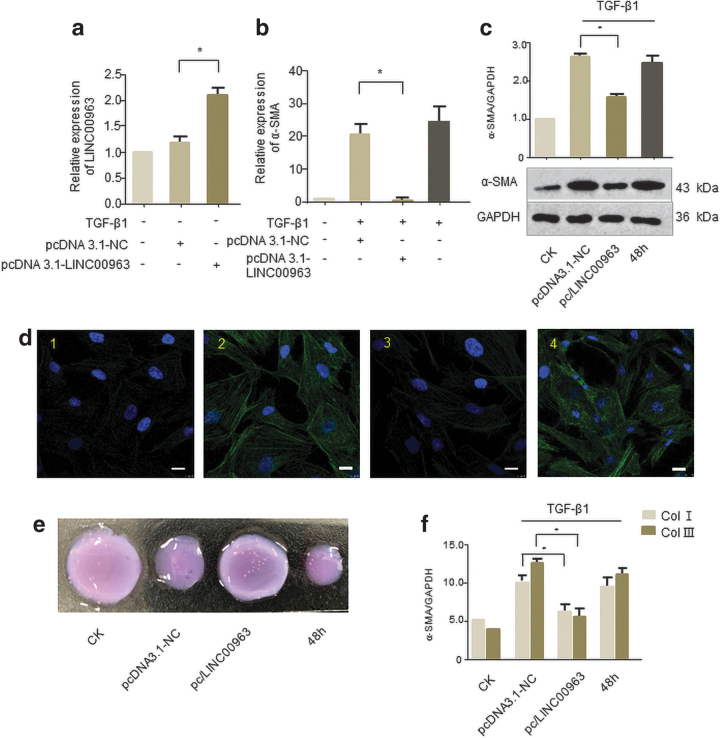
Overexpression of LINC00963 repressed human corneal stromal cells to transdifferentiate into myofibroblasts. **(a)** Human corneal stromal cells were transfected with pcDNA3.1/LINC00963 or pcDNA3.1/NC for 24 h. The expression levels of LINC00963 were detected by qRT-PCR. **(b–d)** Overexpression of LINC00963 reversed TGF-β1-induced transdifferentiation of human corneal stromal cells to myofibroblasts. Cells were transfected with pcDNA3.1/LINC00963 or pcDNA3.1/NC for 24 h and treated with 2 ng/mL TGF-β1 for 24 or 48 h. **(b)** The expression levels of α-SMA mRNA were detected by qRT-PCR (24 h). **(c)** Western blot (48 h) was used to detect α-SMA protein expression. **(d)** Representative immunofluorescence pictures. Cells were stained with DAPI to visualize the nucleus (*blue*), and anti-α-SMA-antibody conjugated to FITC to visualize α-SMA (*green*) as marker for myofibroblast differentiation (1: CK; 2: pcDNA 3.1-NC+TGF-β1 (48 h); 3: pcDNA 3.1-LINC00963+TGF-β1 (48 h); 4: TGF-β1 (48 h); scale bars = 10 μm). **(e, f)** Corneal stromal cells were transfected with pcDNA 3.1-LINC00963 or pcDNA 3.1-NC and then stimulated with TGF-β1 for 48 h. Untreated cells are used as blank control, and only TGF-β1-stimulated cells worked as positive control. **(e)** For contractility assays, cells after treatment were seeded into collagen gels and gel contraction was assessed after 48 h. **(f)** For collagen secretion assay, ELISA was used to analyze the secretion of collagen I and III in cell culture supernatants of each group. *p < 0.05. ELISA, enzyme-linked immunosorbent assay; NC, negative control. Color images are available online.

To assess whether LINC00963 was critical for myofibroblast function, we constructed LINC00963 overexpression plasmid and analyzed changes in the expression of α-SMA, a common marker of myofibroblast. qRT-PCR demonstrated that LINC00963 levels increased twofold compared to NC plasmids (Fig. 3a). And α-SMA mRNA and protein levels were upregulated in TGF-β1-stimulated human corneal stromal cells, but significantly decreased in LINC00963 enforced myofibroblasts compared with NC (Fig. 3b–d). Next, three-dimensional collagen gels were used in corneal stromal cell contraction studies. We can see that overexpression of LINC00963 can significantly reverse the increase of cell contractility treated by TGF-β1 (Fig. 3e). Moreover, LINC00963 can also reverse the collagen I (Col I) and Col III secretion caused by TGF-β1 (Fig. 3f).

### LINC00963 directly regulates the expression of miR-143-3p

**FIG. 4.**
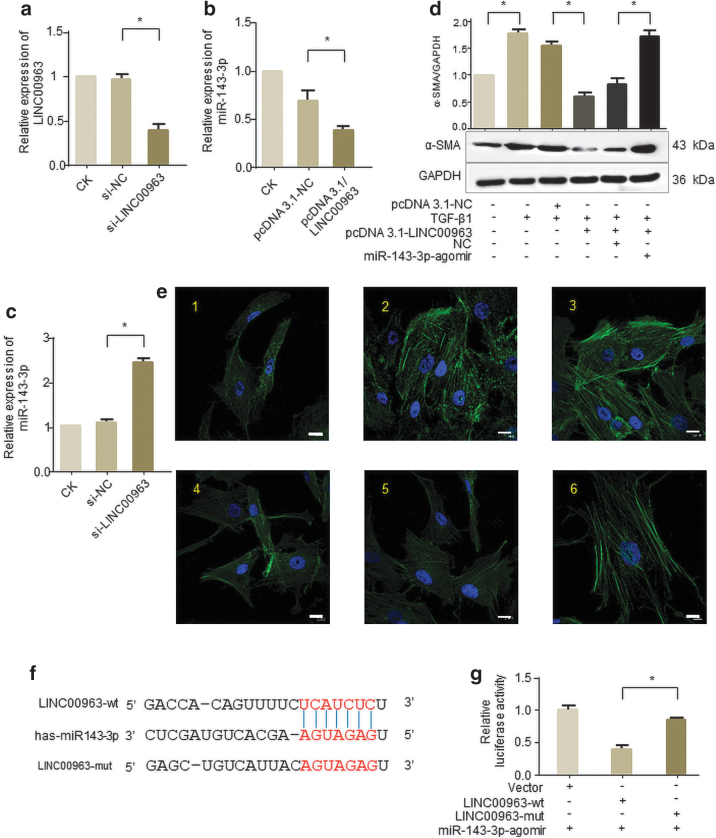
LINC00963 is physically associated with miR-143-3p. Cells were transfected with pcDNA3.1/LINC00963 or siRNA targeting LINC00963 for 24 h. **(a)** Silencing efficiency of si-LINC00963. **(b, c)** miR-143-3p levels were assessed by qRT-PCR and normalized to U6 mRNA levels. **(d)** Enforced expression of LINC00963 decreased the sensitivity of human corneal stromal cells to TGF-β1 (2 ng/mL) treatment, which was abolished by overexpression of miR-143-3p. **(e)** Representative images of confocal microscope show that enforced LINC00963 abated the TGF-β1-induced human corneal stromal cell–myofibroblast transition, which was abolished by overexpression of miR-143-3p (scale bars = 10 μm). **(f)** Schematic representation of the predicted binding site of miR-143-3p on LINC00963 transcript. **(g)** Luciferase activity in human corneal stromal cells cotransfected with miR-143-3p and luciferase reporters containing nothing (vector), LINC00963 (LINC00963-wt), or mutant transcript (LINC00963-mut). Data are presented as the relative ratio of firefly luciferase activity to Renilla luciferase activity. Color images are available online.

Since one of the functional mechanisms of lncRNAs is to act as miRNA sponge (Song *et al.*, 2017; YiRen *et al.*, 2017), we applied Starbase website to predict the downstream target miRNA of LINC00963. Finally, we selected miR-143-3p to verify their interactions, which has been reported to be linked with heart fibrosis (Li *et al.*, 2019). However, the role of miR-143-3p in corneal fibrosis still remains to be explored. We found that enforced LINC00963 reduced miR-143-3p level in human corneal stromal cells (Fig. 4a). In addition, downregulation of LINC00963 increased the expression level of miR-143-3p (Fig. 4b).

Then, we examined whether LINC00963 attenuated corneal fibrosis through sponging miR-143-3p. Overexpression of LINC00963 inhibited TGF-β1 induced the high expression of α-SMA, while forced expression of miR-143-3p could abrogate this inhibition (Fig. 4c). Representative images indicated that human corneal stromal cells are slim and have weak fluorescence expression, but cells become fat and have stronger fluorescence with TGF-β1 treatment. Overexpression of LINC00963 inhibited TGF-β1 induced this change, while forced expression of miR-143-3p abolishes this inhibition (Fig. 4d).

To further confirm the endogenous direct binding between miR-143-3p and LINC00963, we constructed luciferase reporters containing the 200-nt of 3′ LINC00963, which includes wild-type (wt) or mutated miR-143-3p binding sites (Fig. 4e). It shows that overexpression of miR-143-3p inhibited the luciferase activities of the wt reporter vector but not empty or mutant ones in human corneal stromal cells (Fig. 4f), suggesting that LINC00963 may function as miR-143-3p sponge in human corneal stromal cells.

### miR-143-3p facilitated human corneal stromal cells transdifferentiating into myofibroblasts

**FIG. 5.**
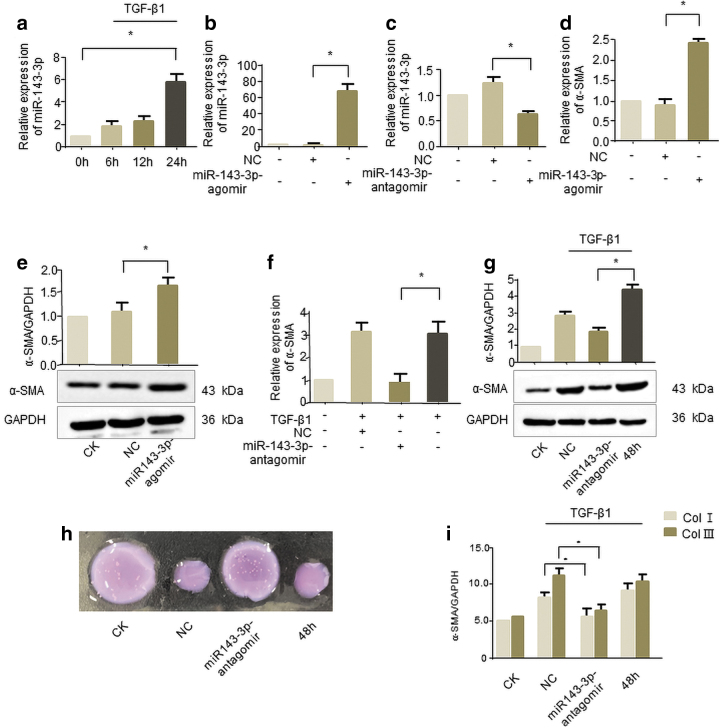
miR-143-3p promotes human corneal stromal cells transdifferentiating into myofibroblasts **(a)** Human corneal stromal cells were treated with 2 ng/mL TGF-β1, and then the expression level of miR-143-3p was measured at the indicated time. **(b)** Transfection with agomir miR-143-3p to upregulating the expression level of miR-143-3p in human corneal stromal cells. Experimental setting blank control group and NC group. Cells were transfected with miR-143-3p agomir or NC for 24 h. The expression level of miR-143-3p was detected using qRT-PCR. **(c)** Knockdown of endogenous miR-143-3p was performed with miR-143-3p antagomir. Cells were transfected with miR-143-3p antagomir or NC for 24 h. The expression level of miR-143-3p was detected using qRT-PCR. **(d, e)** Overexpression of miR-143-3p promoted the expression level of fibrosis marker in α-SMA human corneal stromal cells. Cells were transfected with miR-143-3p agomir or NC for 24 h and 48 h. qRT-PCR (24 h) and western blot (48 h) were used to analyze the expression level of α-SMA. **(f, g)** Knockdown of miR-143-3p eliminated the increase of α-SMA induced by TGF-β1 in human corneal stromal cells. Cells were transfected with miR-143-3p antagomir or NC for 24 h and treated with 2 ng/mL TGF-β1 for 24 and 48 h. qRT-PCR (24 h) and western blot (48 h) were used to analyze the expression level of α-SMA. Data are expressed as the mean ± SEM; *n* = 3 experiments. Corneal stromal cells were transfected with miR-143-3p antagomir or NC and then stimulated with TGF-β1 for 48 h. Nonstimulated cells are used as blank control, and only TGF-β1-stimulated cells worked as positive control. **(h)** For contractility assays, cells after treatment were seeded into collagen gels and gel contraction was assessed after 48 h. **(i)** For collagen secretion assay, ELISA was used to analyze the secretion of collagen I and III in cell culture supernatants of each group. *p < 0.05. Color images are available online.

Then we investigated whether miR-143-3p participated in corneal fibrosis formation. As shown in Figure 5a, miR-143-3p expression was gradually upregulated under TGF-β1 treatment in human corneal stromal cells. Next, cells were transfected with miR-143-3p agomir and antagomir, respectively. And qRT-PCR demonstrated that miR-143-3p levels significantly increased (Fig. 5b) or decreased (Fig. 5c) compared to the NC. The miR-143-3p agomir efficiently promoted the expression of fibrosis marker α-SMA in the level of transcription (Fig. 5d) and translation (Fig. 5e), respectively. Meanwhile, miR-143-3p antagomir can eliminate the rise of α-SMA in TGF-β1 treatment human corneal stromal cells (Fig. 5f, g).

Consistent with the α-SMA expression, we observed that miR-143-3p antagomir not only reverses the increase in cell contraction (Fig. 5h) but also reverses Col I and Col III secretion caused by TGF-β1 (Fig. 5i). These results indicate the involvement of miR-143-3p in TGF-β1 treated human corneal stromal cells, which can facilitate the corneal fibrosis.

## Discussion

The formation of corneal fibrosis scar can cause corneal blindness, which is the second blinding ocular disease in the world (Flaxman *et al.*, 2017). The process of corneal fibrosis scar formation is very complicated, mainly because TGF-β1 secreted from the wound area after corneal injury promotes the transformation of corneal stromal cells into myofibroblasts, which can be identified by its specific marker α-SMA (Blalock *et al.*, 2003; Wilson, 2012).

However, there is no effective treatment for visual loss caused by corneal fibrosis so far. There are still no Drug Administration approved drugs that specifically reduce the expression of corneal fibrosis related genes. Mitomycin C is often applied in some ocular surgeries, but it has side effects such as death of endothelial cells (Zhao *et al.*, 2008), stromal melting, and conjunctival thinning (Raviv *et al.*, 2000; de la Fuente *et al.*, 2008).

Gene therapy has been a research hotspot in recent years, and it is more effective than traditional drugs or antibody therapy. siRNA target drugs have already been used for treatment of ocular diseases. For example, bevasiranib targets the VEGF gene to reduce VEGF production and is used to treat age-related macular degeneration (Garba and Mousa, 2010). Therefore, it is urgent to find a gene therapy for corneal fibrosis scar.

As new therapeutic approach for fibrotic diseases such as cardiac fibrosis (Leisegang, 2018), pulmonary fibrosis (Lu *et al.*, 2018), and renal fibrosis (Wang *et al.*, 2018), lncRNA-based gene therapies have emerged recently. However, the function of lncRNAs in human corneal fibrosis still requires a great deal of exploration.

To identify those lncRNAs that may be involved in the differentiation of human corneal stromal cells, we narrowed down our target range by searching extensive literature. In that TGF-β1 can effectively promote the transdifferentiation of cells into myofibroblasts (Knuppel *et al.*, 2018; Chen *et al.*, 2019). Therefore, 2 ng/mL TGF-β1 was used to stimulate human corneal stromal cells that transdifferentiate to construct a cell model of corneal fibrosis scars and detected the expression of α-SMA by qRT-PCR and western blot (compared with the control group, the expression increased by more than two times) to verify the success of the model. The results showed that the expression of α-SMA was significantly upregulated after TGF-β1 stimulated for 24 h (transcription level) and 48 h (translation level).

The above experiments show that 2 ng/mL TGF-β1 can induce the transdifferentiation of corneal stromal cells into myofibroblasts, which proves that the cell model of corneal fibrosis scar has been successfully constructed. Then the expression level of lncRNAs that may be involved in fibrosis regulation in the literature was detected in this cell model.

LINC00963 was reported to promote melanoma malignant progression by elevating expression of NACC1 through inhibiting miR-608 (Jiao *et al.*, 2018). It also has function in renal interstitial fibrosis and oxidative stress of chronic renal failure using the forkhead box O (FoxO) signaling pathway (Chen *et al.*, 2018).

To date, there was no research uncovering its role in corneal fibrosis. Our experimental evaluation of candidate lncRNAs showed that LINC00963 is significantly downregulated in TGF-β1-induced corneal myofibroblasts in a time-dependent manner, which may be a signal that it can act on the formation of corneal fibrosis scars. Moreover, overexpression of LINC00963 could eliminate the increase of α-SMA caused by TGF-β1. We also observed a strong decrease in myofibroblast contractility and decreased secretion of collagen I and III, revealing a novel mechanism for regulating corneal scars.

lncRNA acts as miRNA sponge, which can disrupt the binding of miRNA to its target mRNA, which consequently promotes mRNA translation. For example, lncRNA-MIAT can upregulate CD47 expression by inhibiting miR-149-5p expression in advanced atherosclerosis (Ye *et al.*, 2019). LINC00346 inhibits BRD4 expression to promote pancreatic cancer growth and gemcitabine resistance, primarily by sponging miR-188-3p (Shi *et al.*, 2019).

In this context, we found that LINC00963 might act as a ceRNA for miR-143-3p using the bioinformatics program Starbase. This view has also been verified by our *in vitro* results. In addition, miR-143-3p was reported to promote human cardiac fibrosis by targeting sprouty3 after myocardial infarction (Li *et al.*, 2019) and promote Col III expression in interstitial fibroblasts (Naito *et al.*, 2014), which provides the probability of its role in corneal fibrosis. Our studies confirmed the findings that LINC00963 could directly bind to the miR-143-3p and suppressed its expression, thereby reversing the effect of miR-143-3p on promoting corneal fibrosis.

In summary, our study identified a lncRNA LINC00963 that inhibits the development and progression of corneal fibrosis. We demonstrated the relationship between LINC00963 and miR-143-3p in TGF-β1-induced corneal fibrosis. Our study may provide theoretical support for a promising treatment strategy for corneal fibrosis suggesting the use of LINC00963 alone or in combination with other anti-scar or inflammatory drugs. However, the involvement of miRNAs and/or molecules in ceRNA networks involving LINC00963 are still next issues to be addressed. Taken together, our research provides new ideas and theoretical support for the treatment of corneal fibrosis, and LINC00963/miR-143-3p axis may provide promising therapeutic approach for the prevention and treatment of corneal fibrosis.

## References

[B1] Abdel-Al, A., El-Ahwany, E., Zoheiry, M., Hassan, M., Ouf, A., Abu-Taleb, H., *et al.* (2018). miRNA-221 and miRNA-222 are promising biomarkers for progression of liver fibrosis in HCV Egyptian patients. Virus Res 253**,** 135–139. 29932949 10.1016/j.virusres.2018.06.007

[B2] Bai, Y.H., Lv, Y., Wang, W.Q., Sun, G.L., and Zhang, H.H. (2018). LncRNA NEAT1 promotes inflammatory response and induces corneal neovascularization. J Mol Endocrinol 61**,** 231–239. 30328354 10.1530/JME-18-0098

[B3] Blalock, T.D., Duncan, M.R., Varela, J.C., Goldstein, M.H., Tuli, S.S., Grotendorst, G.R., *et al.* (2003). Connective tissue growth factor expression and action in human corneal fibroblast cultures and rat corneas after photorefractive keratectomy. Invest Ophthalmol Vis Sci 44**,** 1879–1887. 12714619 10.1167/iovs.02-0860

[B4] Chen, W., Zhang, L., Zhou, Z.Q., Ren, Y.Q., Sun, L.N., Man, Y.L., *et al.* (2018). Effects of long non-coding RNA LINC00963 on renal interstitial fibrosis and oxidative stress of rats with chronic renal failure via the foxo signaling pathway. Cell Physiol Biochem 46**,** 815–828. 29627834 10.1159/000488739

[B5] Chen, Y.J., Huang, S.M., Tai, M.C., Chen, J.T., and Liang, C.M. (2019). Glucosamine impedes transforming growth factor β1-mediated corneal fibroblast differentiation by targeting Krüppel-like factor 4. J Biomed Sci **26,** 72. 10.1186/s12929-019-0566-1 PMC6784344 31597574

[B6] de la Fuente, M., Seijo, B., and Alonso, M.J. (2008). Bioadhesive hyaluronan-chitosan nanoparticles can transport genes across the ocular mucosa and transfect ocular tissue. Gene Ther 15**,** 668–676. 18305575 10.1038/gt.2008.16

[B7] Dvashi, Z., Sar Shalom, H., Shohat, M., Ben-Meir, D., Ferber, S., Satchi-Fainaro, R., *et al.* (2014). Protein phosphatase magnesium dependent 1A governs the wound healing-inflammation-angiogenesis cross talk on injury. Am J Pathol 184**,** 2936–2950. 25196308 10.1016/j.ajpath.2014.07.022

[B8] Flaxman, S.R., Bourne, R.R.A., Resnikoff, S., Ackland, P., Braithwaite, T., Cicinelli, M.V., *et al.* (2017). Global causes of blindness and distance vision impairment 1990–2020: a systematic review and meta-analysis. Lancet Global Health 5:e1221–e1234. 29032195 10.1016/S2214-109X(17)30393-5

[B9] Funari, V.A., Winkler, M., Brown, J., Dimitrijevich, S.D., Ljubimov, A.V., and Saghizadeh, M. (2013). Differentially expressed wound healing-related microRNAs in the human diabetic cornea. PLoS One **8,** e84425. 10.1371/journal.pone.0084425 PMC3869828 24376808

[B10] Garba, A.O., and Mousa, S.A. (2010). Bevasiranib for the treatment of wet, age-related macular degeneration. Ophthalmol Eye Dis 2**,** 75–83. 23861616 10.4137/OED.S4878 PMC3661434

[B11] Huang, J., Li, Y.J., Liu, J.Y., Zhang, Y.Y., Li, X.M., Wang, L.N., *et al.* (2015). Identification of corneal neovascularization-related long noncoding RNAs through microarray analysis. Cornea 34**,** 580–587. 25747163 10.1097/ICO.0000000000000389

[B12] Hwang, Y.H., Jung, S.A., Lyu, J., Kim, Y.Y., and Lee, J.H. (2019). Transforming Growth Factor-beta1-induced human subconjunctival fibrosis is mediated by MicroRNA 143/145 expression. Invest Ophthalmol Vis Sci 60**,** 2064–2071. 31081880 10.1167/iovs.19-26797

[B13] Jiao, H., Jiang, S., Wang, H., Li, Y., and Zhang, W. (2018). Upregulation of LINC00963 facilitates melanoma progression through miR-608/NACC1 pathway and predicts poor prognosis. Biochem Biophys Res Commun 504**,** 34–39. 30180950 10.1016/j.bbrc.2018.08.115

[B14] Kawasaki, N., Miwa, T., Hokari, S., Sakurai, T., Ohmori, K., Miyauchi, K., *et al.* (2018). Long noncoding RNA NORAD regulates transforming growth factor-beta signaling and epithelial-to-mesenchymal transition-like phenotype. Cancer Sci 109**,** 2211–2220. 29722104 10.1111/cas.13626 PMC6029837

[B15] Knuppel, L., Heinzelmann, K., Lindner, M., Hatz, R., Behr, J., Eickelberg, O., *et al.* (2018). FK506-binding protein 10 (FKBP10) regulates lung fibroblast migration via collagen VI synthesis. Respir Res **19,** 67. 10.1186/s12931-018-0768-1 PMC5909279 29673351

[B16] Kugel, J.F., and Goodrich, J.A. (2012). Non-coding RNAs: key regulators of mammalian transcription. Trends Biochem Sci 37**,** 144–151. 22300815 10.1016/j.tibs.2011.12.003 PMC3323709

[B17] Leisegang, M.S. (2018). LET's sponge: how the lncRNA PFL promotes cardiac fibrosis. Theranostics 8**,** 874–877. 29463987 10.7150/thno.23364 PMC5817098

[B18] Li, C., Li, J., Xue, K., Zhang, J., Wang, C., Zhang, Q., *et al.* (2019). MicroRNA-143-3p promotes human cardiac fibrosis via targeting sprouty3 after myocardial infarction. J Mol Cell Cardiol 129**,** 281–292. 30878395 10.1016/j.yjmcc.2019.03.005

[B19] Li, X., Zhou, H., Tang, W., Guo, Q., and Zhang, Y. (2015). Transient downregulation of microRNA-206 protects alkali burn injury in mouse cornea by regulating connexin 43. Int J Clin Exp Pathol 8**,** 2719–2727. 26045777 PMC4440086

[B20] Lu, Q., Guo, Z., Xie, W., Jin, W., Zhu, D., Chen, S., *et al.* (2018). The lncRNA H19 mediates pulmonary fibrosis by regulating the miR-196a/COL1A1 axis. Inflammation 41**,** 896–903. 29411215 10.1007/s10753-018-0744-4

[B21] Milani, B.Y., Milani, F.Y., Park, D.W., Namavari, A., Shah, J., Amirjamshidi, H., *et al.* (2013). Rapamycin inhibits the production of myofibroblasts and reduces corneal scarring after photorefractive keratectomy. Invest Ophthalmol Vis Sci 54**,** 7424–7430. 24106124 10.1167/iovs.13-12674 PMC3828043

[B22] Naito, Y., Sakamoto, N., Oue, N., Yashiro, M., Sentani, K., Yanagihara, K., *et al.* (2014). MicroRNA-143 regulates collagen type III expression in stromal fibroblasts of scirrhous type gastric cancer. Cancer Sci 105**,** 228–235. 24283360 10.1111/cas.12329 PMC4317817

[B23] Park, J.K., Peng, H., Yang, W., Katsnelson, J., Volpert, O., and Lavker, R.M. (2017). miR-184 exhibits angiostatic properties via regulation of Akt and VEGF signaling pathways. FASEB J 31**,** 256–265. 27825105 10.1096/fj.201600746R PMC5161520

[B24] Peng, H., Wan, L.Y., Liang, J.J., Zhang, Y.Q., Ai, W.B., and Wu, J.F. (2018). The roles of lncRNA in hepatic fibrosis. Cell Biosci **8,** 63. 10.1186/s13578-018-0259-6 PMC6282372 30534359

[B25] Qi, D., Li, J., Que, B., Su, J., Li, M., Zhang, C., *et al.* (2016). Long non-coding RNA DBCCR1-003 regulate the expression of DBCCR1 via DNMT1 in bladder cancer. Cancer Cell Int **16,** 81. 10.1186/s12935-016-0356-8 PMC5069854 27777512

[B26] Ratuszny, D., Gras, C., Bajor, A., Borger, A.K., Pielen, A., Borgel, M., *et al.* (2015). miR-145 is a promising therapeutic target to prevent cornea scarring. Hum Gene Ther 26**,** 698–707. 26165705 10.1089/hum.2014.151

[B27] Raviv, T., Majmudar, P.A., Dennis, R.F., and Epstein, R.J. (2000). Mytomycin-C for post-PRK corneal haze. J Cataract Refract Surg 26**,** 1105-1106. 11041724 10.1016/s0886-3350(00)00625-8

[B28] Shi, W., Zhang, C., Ning, Z., Hua, Y., Li, Y., Chen, L., *et al.* (2019). Long non-coding RNA LINC00346 promotes pancreatic cancer growth and gemcitabine resistance by sponging miR-188-3p to derepress BRD4 expression. J Exp Clin Cancer Res **38,** 60. 10.1186/s13046-019-1055-9 PMC6366022 30728036

[B29] Song, Y.X., Sun, J.X., Zhao, J.H., Yang, Y.C., Shi, J.X., Wu, Z.H., *et al.* (2017). Non-coding RNAs participate in the regulatory network of CLDN4 via ceRNA mediated miRNA evasion. Nat Commun **8,** 289. 10.1038/s41467-017-00304-1 PMC5561086 28819095

[B30] Tao, H., Zhang, J.G., Qin, R.H., Dai, C., Shi, P., Yang, J.J., *et al.* (2017). LncRNA GAS5 controls cardiac fibroblast activation and fibrosis by targeting miR-21 via PTEN/MMP-2 signaling pathway. Toxicology 386**,** 11–18. 28526319 10.1016/j.tox.2017.05.007

[B31] Tomasek, J.J., Gabbiani, G., Hinz, B., Chaponnier, C., and Brown, R.A. (2002). Myofibroblasts and mechano-regulation of connective tissue remodelling. Nat Rev Mol Cell Biol 3**,** 349–363. 11988769 10.1038/nrm809

[B32] Wang, J., Pang, J., Li, H., Long, J., Fang, F., Chen, J., *et al.* (2018). lncRNA ZEB1-AS1 was suppressed by p53 for renal fibrosis in diabetic nephropathy. Mol Ther Nucl Acids 12**,** 741–750. 10.1016/j.omtn.2018.07.012 PMC6095953 30121551

[B33] Wei, P., Xie, Y., Abel, P.W., Huang, Y., Ma, Q., Li, L., *et al.* (2019). Transforming growth factor (TGF)-β1-induced miR-133a inhibits myofibroblast differentiation and pulmonary fibrosis. Cell Death Dis **10,** 670. 10.1038/s41419-019-1873-x PMC6739313 31511493

[B34] Wilson, S.E. (2012). Corneal myofibroblast biology and pathobiology: generation, persistence, and transparency. Exp Eye Res 99**,** 78–88. 22542905 10.1016/j.exer.2012.03.018 PMC3367126

[B35] Wu, J.H., Tian, X.Y., An, Q.M., Guan, X.Y., and Hao, C.Y. (2018). LINC00963 promotes hepatocellular carcinoma progression by activating PI3K/AKT pathway. Eur Rev Med Pharmacol Sci 22**,** 1645–1652. 29630107 10.26355/eurrev_201803_14574

[B36] Yan, B., Yao, J., Liu, J.Y., Li, X.M., Wang, X.Q., Li, Y.J., *et al.* (2015). lncRNA-MIAT regulates microvascular dysfunction by functioning as a competing endogenous RNA. Circ Res 116**,** 1143–1156. 25587098 10.1161/CIRCRESAHA.116.305510

[B37] Yang, X., Teng, Y., Hou, N., Fan, X., Cheng, X., Li, J., *et al.* (2011). Delayed re-epithelialization in Ppm1a gene-deficient mice is mediated by enhanced activation of Smad2. J Biol Chem 286**,** 42267–42273. 21990361 10.1074/jbc.M111.292284 PMC3234921

[B38] Ye, Z.M., Yang, S., Xia, Y.P., Hu, R.T., Chen, S., Li, B.W., *et al.* (2019). LncRNA MIAT sponges miR-149-5p to inhibit efferocytosis in advanced atherosclerosis through CD47 upregulation. Cell Death Dis **10,** 138. 10.1038/s41419-019-1409-4 PMC6372637 30755588

[B39] YiRen, H., YingCong, Y., Sunwu, Y., Keqin, L., Xiaochun, T., Senrui, C., *et al.* (2017). Long noncoding RNA MALAT1 regulates autophagy associated chemoresistance via miR-23b-3p sequestration in gastric cancer. Mol Cancer **16,** 174. 10.1186/s12943-017-0743-3 PMC5699172 29162158

[B40] Zhang, Y., Zhang, T., Ma, X., and Zou, J. (2017). Subconjunctival injection of antagomir-21 alleviates corneal neovascularization in a mouse model of alkali-burned cornea. Oncotarget 8**,** 11797–11808. 28052006 10.18632/oncotarget.14370 PMC5355305

[B41] Zhao, L.Q., Wei, R.L., Ma, X.Y., and Zhu, H. (2008). Effect of intraoperative mitomycin-C on healthy corneal endothelium after laser-assisted subepithelial keratectomy. J Cataract Refract Surg 34**,** 1715–1719. 18812123 10.1016/j.jcrs.2008.06.016

[B42] Zhao, X., Sun, J., Chen, Y., Su, W., Shan, H., Li, Y., *et al.* (2018). lncRNA PFAR promotes lung fibroblast activation and fibrosis by targeting miR-138 to regulate the YAP1-twist axis. Mol Ther 26**,** 2206–2217. 30025992 10.1016/j.ymthe.2018.06.020 PMC6127506

[B43] Zhou, Y., Yin, L., Li, H., Liu, L.H., and Xiao, T. (2019). The LncRNA LINC00963 facilitates osteosarcoma proliferation and invasion by suppressing miR-204-3p/FN1 axis. Cancer Biol Ther 20**,** 1141–1148. 30975024 10.1080/15384047.2019.1598766 PMC6605988

[B44] Zou, T., Jaladanki, S.K., Liu, L., Xiao, L., Chung, H.K., Wang, J.Y., *et al.* (2016). H19 long noncoding RNA regulates intestinal epithelial barrier function via microRNA 675 by interacting with RNA-binding protein HuR. Mol Cell Biol 36**,** 1332–1341. 26884465 10.1128/MCB.01030-15 PMC4836219

